# The cultural change narrative as a core component of therapeutic change

**DOI:** 10.3389/fpsyt.2023.1149984

**Published:** 2023-10-06

**Authors:** Astrid Hermann Tobiassen, Thea Sundal, Erik Stänicke, Espen Jan Folmo

**Affiliations:** ^1^Akershus University Hospital, Lørenskog, Norway; ^2^Helgeland Hospital, Sandnessjøen, Norway; ^3^Department of Psychology, Faculty of Social Sciences, University of Oslo, Oslo, Norway; ^4^INSEAD, Bd de Constance, Fontainebleau, France

**Keywords:** therapeutic change, change narrative, personality disorder, qualitative analysis, Mentalization-based treatment, Dialectical behavior therapy, Interpretative Phenomenological Analysis

## Abstract

**Introduction:**

Research indicates a similar effect of Mentalization-based treatment (MBT) and Dialectical behavior therapy (DBT) for borderline personality disorder (BPD). However, there is a paucity in studies investigating the change narrative received from and developed in these treatments. The aim of the present study is to investigate similarities and differences in the change narratives provided by MBT and DBT, and how these narratives reflect the rationale, explanations, and procedures of the provided treatment.

**Methods:**

The study is a qualitative analysis of seven interviews conducted by the authors. Three of the participants had received MBT, and four of the participants had received DBT. This study presents an Interpretative Phenomenological Analysis (IPA) of the change narratives received in two specialized treatments for BPD.

**Results:**

The main findings from the IPA were that the change narratives described by the participants reflected the treatment they received. The DBT participants highlighted explicit learning of tools and techniques, with predictable and safe therapists. In contrast, the MBT participants emphasized a long-lasting process of exploring to create procedural learning with therapists who followed their lead.

**Discussion:**

The participants’ stories of change shed light on how a change narrative was developed, and therefore how the rationale, explanations and procedures were conveyed differently by MBT and DBT.

## Introduction

“Everybody has won, and all must have prizes” was the Dodo bird’s verdict in Lewis Carroll’s *Alice’s Adventures in Wonderland* (1). The Dodo bird verdict in psychotherapy research refers to the observation that a wide range of schools of psychotherapy seem to be equally effective (2). This has later been largely revisited (3–5). One way of understanding the lack of differences is that all psychological treatments have some shared healing ingredients [(6), p. 39]. The working alliance and the therapist factor are common factors that have been widely studied (7–14). While the therapeutic alliance is the most robust predictor of psychotherapeutic healing, explaining around 7.5% of the variance in outcomes (8), and no statistical differences can be seen between *bona fide* treatments, research suggests that the common factors unfold differently in different treatments (15–19). Research suggests that patients who have received cognitive behavioral therapy and psychodynamic therapy will have qualitatively different descriptions of these experiences (20). MBT and DBT are two specialized treatments for borderline personality disorder. DBT has roots in the cognitive tradition (21), while MBT is a psychodynamic inspired treatment (22). They have several similarities, such as the length of treatment, the combination of individual therapy and groups, and they produce similar results on outcome measures (23–27). However, the specific ingredients which constitute the treatments will inevitably provide patients who receive MBT or DBT with different experiences of change.

In *Persuasion and Healing* (1993), Frank and Frank proposed four effective features shared by most healing rituals across cultures and across time. One of these was “a rationale, conceptual scheme, or myth, that provides a plausible explanation for the patient’s symptoms and prescribes a ritual or procedure for resolving them” [(6), p. 42]. This common factor could shed light on how elements found in every psychotherapeutic treatment, put in context of their specific ingredients, provide patients with different experiences of change. In Western societies psychotherapy could be understood as an acknowledged healing ritual for psychological suffering, trusted and believed in the culture (6). Furthermore, cultural recognition of the healing rituals provides the necessary social legitimacy to the therapeutic rituals or procedures, and to the rationale provided in the therapeutic setting (28). Each therapeutic method will also provide their own specific culture which will be transmitted to the patient. This may happen through the specific language used to conceptualize the healing myth, and the specific explanations for the patient’s problems, as well as through the unique rituals put forth as necessary for resolving them.

Laska et al. (29) have proposed a common factor (CF) approach that seems to be strongly inspired by Frank and Frank (6). This approach includes an entrusting therapeutic setting, a provided rationale accepted by the patient, a culturally embedded explanation for the disorder, which is being treated, an emotional bond in the therapeutic relationship, and therapeutic procedures. Wampold and Imel (30) proposed that if therapists can provide a believable rationale that is accepted, expectations connected to the positive effects of the treatment may arise. Frank and Frank (6) tied the provision of a rationale to the therapist’s ability to combat demoralization and inspire hope in the patient. Several decades of psychotherapy research have suggested that the patient’s belief in the treatment, in addition to motivation and expectations, is associated with positive outcomes (6, 30–33). Furthermore, the importance of expectations and hope could be illuminated by studies on the placebo effect (6, 33–36).

BPD is defined by DSM-V as “a pervasive pattern of instability of interpersonal relationships, self-image, and affects, and marked impulsivity, beginning by early adulthood and present in a variety of contexts” (37). BPD is a severe psychiatric disorder, traditionally seen as difficult to treat, and is characterized by pathology that affects the patient, relatives, and the society (38, 39). Twin studies have shown that a genetic predisposition to emotional dysregulation combined with a non-supporting environment can lead to the development of BPD (40–42). The point prevalence of personality disorders in general is estimated to be around 10%, and for BPD specifically 1.5% (43, 44). Patients with BPD share some core characteristics. Some of these are suicidal thoughts and attempts, and self-harm. Studies have reported a 8–10% suicide rate among BPD patients, and a lifetime prevalence of 3–4 attempts (45–48). While pharmacological interventions on BPD show no more than moderate effect, over half a dozen manualized treatment methods have been empirically validated for treatment of the disorder (25, 49). However, the evidence indicates that some specific therapies are superior to usual care (27, 50). DBT and MBT are the two most commonly used treatments for BPD in Norway (39).

MBT is a manualized treatment developed by Anthony Bateman and Peter Fonagy shown to be efficient for BPD (51–56). The environment in which attachment relationships are formed is crucial for the development of mentalization, and Fonagy and Bateman (57) have postulated that an interaction between disturbances in early development of attachment, and a person’s neurological development are central to the development of BPD. The ability to mentalize can be weakened by insecurities in these attachment relationships (57). Poor mentalization is related to reduced social functioning, low quality relationships and psychopathology in general (58). Bateman and Fonagy (22) have described that the goal of MBT is to restore the ability of mentalization when it is lost, maintain it when it is present, and keep it going when it elsewhere would be lost. A mentalizing modus of organizing subjective experiences is in contrast to pre-mentalizing modi, such as pretend mode and psychic equivalence (39). The proposed mechanism of change is an “exclusive focus on the BPD patients’ current mental state while activating the attachment relationship” [(59), p. 21]. MBT assumes that working with trust in the therapeutic relationship is central to healing (39, 60, 61). Furthermore, epistemic trust is at the core of MBT’s recent placement of social learning at the center in understanding mentalizing (59, 62, 63). Epistemic trust can be defined as a person’s ability to evaluate information from the social world and consider its accuracy and reliability in terms of personal relevance. If so, information is allowed to be incorporated into one’s existing knowledge (64).

Dialectical behavior therapy (DBT) is another manualized treatment for BPD developed by Marsha Linehan. DBT is highly structured and consists of four components: (i) Individual therapy; (ii) Group skills training; (iii) Between session telephone coaching; (iv) A therapist consultation team (65, 66). DBT’s dialectical philosophy emphasizes a synthesis between strategies that promote change and acceptance (21, 66). Important treatment targets are emotional, interpersonal, and behavioral dysregulation, and harmful behavior such as self-harm and suicidal acts (66, 67). DBT’s biosocial theory of BPD suggests that emotional dysregulation, originating from emotional vulnerability, and emotion regulation deficits are core themes of BPD (21).

It has been suggested that individuals with BPD have a stronger activation in response systems for emotions, which can be a consequence of a biological vulnerability as well as events in early childhood. Childhood events like neglect or traumas can lead to changes in the development of neural structures important for emotion regulation (68). As a result of this, it is thought that BPD patients have an emotional system that reacts stronger and faster to cues from the environment than others, which makes them more prone to behavioral and cognitive dysregulation (68). In DBT acquisition, strengthening and generalization of abilities that make the patients more resilient facing emotional dysregulation, stress and difficult relationships are core focus areas (21, 67). Central change mechanisms in DBT are motivating the patients to recognize and accept their affective states, and at the same time encourage them to apply different tools and skills to downregulate intense emotions and handle stressful situations constructively (66, 67). Studies indicate that DBT is an effective treatment of BPD as well as suicidality and self-injurious behavior (65, 67, 69–80).

Both MBT and DBT are therapeutic methods that have been widely studied, both quantitatively and qualitatively. Several of the helpful elements in each method have been identified. However, the way in which the culturally transmitted elements in treatment affect a patient’s story of change has, to our knowledge, been less investigated. In this study, we propose that these elements in sum provide the patient with a cultural change narrative that must be phenomenologically investigated to be understood. As Wilber stated in 2000, “exterior surfaces can be seen, but interior depth must be interpreted” [(81), p. 184]. It seems reasonable to question whether the relational co-creation of an accepted cultural change narrative—including its inherent world view and implied isometrics of interpersonal interacting—may foster qualitative changes currently neglected (despite this common factor theorized at core of psychotherapy).

The aim of the study was to openly investigate how patients who have received MBT or DBT experienced change in the treatments they received. When we looked closer at what the participants spoke about in the interviews, we became more and more interested in the systematically different change narratives they received from MBT and DBT, respectively. Furthermore, we wished to investigate how the similarities and differences in change narratives could be understood in light of the treatments’ rationales, explanations, and procedures.

## Materials and methods

### Method

Interpretative Phenomenological Analysis (IPA) has been used as the fundamental method throughout the study. IPA is a qualitative method particularly suitable when seeking to understand an individual’s experience of their own lived experiences, and the meaning they relate to those experiences (82). The development and framework of IPA is informed by concepts and debates from multiple philosophical traditions which attend to the philosophy of knowledge – phenomenology, hermeneutics, and an idiographic approach (82).

Both the phenomenological and the hermeneutic perspective is essential in IPA, as well as insights from the combination of these two. IPA is phenomenological because it is concerned with exploring a person’s experience in its own terms. Using IPA, the researcher strives to make sense of what the participant is trying to make sense of, namely what has happened, or is happening, to them (83). Hermeneutics’ position in IPA is particularly the idea of double hermeneutics; meaning the participant is trying to make sense of their own experience, and the researcher is trying to make sense of the material the participant puts forward in an interview (82). Hence, the use of double hermeneutics is central for understanding the dynamic process between the material, the researcher, and the participant.

The Norwegian Regional Committees for Medical and Health Research Ethics (reference number: 280677) and National Center for Research Data (reference number: 424892) have approved the study. The participants received written and oral information from the authors about the project and its purposes. All participants signed a letter of consent before the interviews were conducted.

### Participants

The sample consisted of seven participants. Four participants had received DBT and three had received MBT. Six of the participants were female, and one of them male. The participants’ age ranged from the mid-twenties to the mid-forties. According to themselves, all the participants met the inclusion criteria for the project. Inclusion criteria included that they at some point had met the criteria for F60.3 Emotional unstable personality disorder according to ICD-10, they had gone to either DBT or MBT, they ended their course of treatment at least 3 years ago and that they felt they could speak freely of their experience of receiving MBT or DBT. Two of the MBT participants received the regular MBT program, with 3 years in MBT-G and MBT-I, while one of them received a 16 weeklong MBT group, as well as receiving individual therapy. All the DBT participants received the regular DBT program.

### Procedure

Five of the participants were recruited through two different Facebook groups where the invitation letter was posted, and two were recruited by a communications advisor at National Advisory Unit on Personality Psychiatry. Potential participants then contacted one of the authors by e-mail or SMS. This enabled the authors to contact the potential participants by telephone, and further information about the project was given. After the telephone contact, the participants were sent a digital letter of consent they could sign by using an electronic identification system. Five of the interviews were conducted via Zoom, and two were conducted in the premises of the Psychology department of University of Oslo.

The semi-structured interview guide was developed to suit a qualitative in-depth interview. The questions were categorized as *before therapy*, *during therapy,* and *after therapy*. The interview guide was later modified to better suit the IPA framework as it is presented by Smith et al. (82). For instance, to reduce the number of questions in the interview guide, some of the questions were changed to probes that could be used if the main questions were perceived as too broad by the participant. The focus of the interviews was the participants’ lived experience of therapy, in accordance with IPA. To achieve this format of the interviews, the preparations for the interviews were loosely inspired by the Life-mode Interview developed by Haavind (84) as we tried to capture concrete experiences in the participants’ everyday lives. The interviews were conducted during a period of 3 weeks in November 2021.

### Data analysis

The data-analysis consisted of several steps, in line with Smith et al. (82). The first step was reading the interviews several times and listening to the recordings. Attention was particularly paid to the interviews conducted by the other author. The second step consisted of writing summaries for each interview, including repeating ideas, important preliminary codes, as well as the shape and flow of the interviews. The interviewers first discussed these impressions with each other, then later discussed them with two senior researchers (third and fourth author). This process was inspired by the ideas of phenomenology and hermeneutics in the sense that we strived to get close to the participant’s experiences, by reading, rereading, listening, and discussing impressions, but at the same time unavoidably interpreting the participants’ stories in accordance with pre-existing knowledge.

The third step consisted of identifying emerging themes and categories from the material. The selected quotes from the interviews were coded descriptively to emphasize what the participants spoke about in the interviews. At the completion of initial coding, we discussed which emergent themes could be found both within and across the interviews. The analysis across was made between each of the MBT interviews, each of the DBT interviews, and between the MBT and the DBT interviews. This step was strongly inspired by the hermeneutic circle, where the material was pulled apart and put together several times.

The fourth step of the analysis consisted of searching for connections in the material by making sense of the codes and placing them into a hierarchy. As a part of step five, moving to the next case, this process was applied to each interview. Step six consisted of looking for patterns across the cases. The descriptive codes for each interview were clustered into sub-themes, which then were abstracted to themes, and this led us to the meta-themes. Subsequently, the meta-themes, themes, and sub-themes of the DBT interviews and the MBT interviews were made into two separate hierarchies. All the meta- themes in both hierarchies were divided into two topics: Before MBT or DBT and during/after MBT or DBT.

The third and fourth authors were consulted throughout the analysis process to secure validity to our findings. Furthermore, the names of meta-themes, themes and sub-themes were discussed, and some were re- labeled, merged, and moved in a process of trying to make the hierarchies as close to experience as possible. Quotations from the interviews were then selected to illustrate the final themes and sub-themes. The hierarchies are presented in Appendix A, and the quotes selected are presented in the results section below. The Table 1 aims to illustrate the process from quote to meta-theme.

**Table 1:** Example of analysis.

Quote	Preliminary code	Sub-theme	Theme	Meta-theme
*“Learning that it’s not dangerous even though it hurts, it will pass. It helped me a lot when I learned that [the feeling] will always pass. And I had not thought of that before, I was so preoccupied with how painful it was”*	Learning that feelings eventually will pass	Learning about feelings	Learning new ways of understanding myself and others	Explicit learning of a provided approach specific to my struggles

## Results

The data analysis resulted in eight meta-themes distributed on two topics – before treatment and during/after treatment. Four meta-themes organize the analysis of the MBT interviews, and four meta-themes organize the DBT interviews. The meta-themes hierarchically organize associated themes, which in turn organize the sub-themes. The themes and sub-themes are not discussed separately. However, the whole hierarchy of the analysis can be found in Appendix A. The participants are given fictional names when examples and quotes are used. In the following, hierarchy will be presented with selected quotes. Some of the sub-themes will inevitably represent some participants more than they represent others.

### First topic: before treatment

#### First meta-theme DBT: I lacked an understanding of myself, and coped with my struggles destructively

This meta-theme seeks to capture the extent in which the participants felt they were not connected to, or in control of, their inner states, and how they handled these experiences. All four participants described strong emotional intensity and instability, as well as overwhelming emotions. For example, Anna explained: “*It was the experience of being overwhelmed, that came with the emotional instability, when I was tired, shameful, or felt insulted.*” They also talked about emotional chaos and incomprehensive feelings.

As a result of an overwhelming emotional chaos, some of the participants described a desire to change the way they felt, and how they could not do it before treatment. Eva explained, “*I could not get myself up or change my mindset. To get up and out of bed. Life just felt hopeless and over.*” This inability to change her way of thinking made her feel like she could not control what happened to her. Similarly, Anna talked about the lack of control, “*It was very unstable, and I did not know how to make it stop*.” This may imply a belief that with the proper knowledge, feelings can be controlled and changed. Furthermore, it may suggest that Anna learned some tools in DBT that enabled her to control her emotions to a greater extent. All the participants described self-harm as a way of handling these painful emotions that felt out of control. Self-harm became a way of changing focus and relocating their pain as well as a relief of internal pressure. Lisa talked about when and why she would harm herself, “*It was under strong frustration or despair. And it was because [the frustration or despair] got an outlet somewhere. The pain moved from one place to another.*” All four of the participants had a clear picture of how self-harming felt like and the function it had for them, and for all of them it seemed to be a way of gaining a sense of control which they lacked regarding their inner states. All the participants spoke relatively freely about their self-harming experiences.

#### First meta-theme MBT: my life lacked coherence

This meta-theme seeks to capture the participants’ descriptions of their lives before they received MBT, both in terms of symptomatology and coping mechanisms. They all described lives that lacked coherence in terms of the self, understanding of others, and their coping mechanisms. Daniel and Miriam described their everyday functioning as limited. Daniel said, *“A typical day consisted of either sleeping all day or going to the activity center. Very little was happening. Very navel-gazing, one can say,”* and Miriam said, “*Get the kids to kindergarten, go home, lay and stare at the wall.”* All three participants described a chaotic inner state, in one way or another. Miriam described how she withdrew due to her chaotic internal experiences, while Daniel described his life as a rollercoaster, meaning his life was unpredictable and unstable. Amanda described how she managed to keep a façade, with an education and a job she was good at. However, her private life was troublesome. She said, “*I had enormous problems in my personal relationships. I have self- harmed since my early teens, but I hid it from everyone. The self-harm and how I actually was doing.”*

All the participants dealt with the lack of coherence in harmful ways. Miriam developed a severe eating disorder, which became a way of gaining control. Daniel struggled with suicidal ideation and often expressed a wish to die to others when he struggled. He also said, “*I understand now that such expressions [“I just want to go and hang myself”] can trigger reactions in others*,” implying in treatment, he reached an understanding of how his actions could have a direct effect on other people. Daniel mentioned his struggles with self-harm by cutting, Amanda said she struggled with self-harm throughout therapy, and Miriam mentioned she had several suicide-attempts behind her. Neither went further into detail about the circumstances of the self-harm or suicidal behavior.

#### Second meta-theme DBT: my struggles with seeing the situation from an outside perspective

The second meta-theme of DBT concerns how the participants were sensitive to what they believed other peoples’ opinions of them were, and how this led to difficulties in close and less close relationships. Both Eva and Anna talked about how they felt ashamed of what they believed other people thought of their behavior. Eva described how shame led her to withdraw from others: “*If I woke up on a Wednesday, was tired and did not have the energy to go to work, then the shame was too bad for me to go back to work on Thursday.*” They all described how small things could make them feel like people did not like them or care for them, and how painful this could be.

The tendency to interpret other people’s behavior toward them negatively was described by all four participants as a core difficulty. Eva said, “*I often misinterpreted other people. I could believe they were criticizing me when they were just… I do not know… trying to correct me […] I always thought they meant to hurt me, not that they were trying to help.”* This quote describes how easily Eva felt criticized and how her interpretations led to a belief that other people were trying to be unkind. Lisa, Anna, and Sara also described how they would interpret small things, such as a look or a glance, negatively, and how they quickly jumped to the conclusion that people did not like them.

#### Second meta-theme MBT: how my problems with mentalization affected myself and others

This meta-theme of MBT seeks to capture how the participants’ internal pain also affected their relationships. Both Amanda and Daniel had challenges with boundaries. For Amanda, it was also connected to her inability to tell others what she needed and meant. She said, “*It was often something that was bothering me about a person, but I could not tell them. Instead, it became a very forceful reaction, so it became almost impossible to sort it out. In the end, you end up burning all your relationships*.” This may imply that Amanda’s lack of understanding of own inner state made it difficult to know her own and others’ boundaries. Daniel described aggression as one of the only ways to communicate his boundaries to others. He said, “*When something made me angry, I knew it wasn’t necessarily rooted in reality, but it was aggression towards everything and everyone. The whole world, in fact*.” Daniel also struggled with differentiation of affect, he only knew happy and angry. His lack of understanding of his own feelings and own reactions led to internal pain and great struggles in his relationships. The participants also talked about how they had little room internally for others. Misinterpretation was particularly common, and they connected this to their lack of understanding of themselves. Amanda said, “*When you do not know what is happening inside, it gets hard to interpret external signals, and it becomes a mess. And then there might be strong reactions to little stimuli. I misinterpreted*.” Daniel connected his lack of mentalization to how he over-analyzed his environment. Miriam also talked about how she was afraid of what other people thought about her. That is, her fantasies about what an imagined other might have thought of her struggles if they knew. She said, “*That shame, the defeat you carry, you cannot tell others because people are going to laugh. You go around thinking, no one feels this way, and you must try … society expects you to be normal.”* This fear became less prominent during and after therapy, which may imply that an increased capability to mentalize not only affected her close relationships, but also how she interacted with her environment.

### Second topic: during and after treatment

#### First meta-theme DBT: explicit learning of a provided approach specific to my struggles

The second topic is concerned with the participants’ experience of change during and after therapy. One central aspect of change the participants spoke of was how they learned to look at a situation more objectively. They explained how they learned to take an outside perspective of a situation, rather than to rely on their own thoughts, feelings, and interpretations. This made them see that their interpretations of situations did not necessarily coincide with what was happening, and this provided new meaning to situations. In the interview Lisa described how DBT dramatically changed the way she viewed the world and her own contributions.

Lisa: It was almost like a … paradigm shift. Because I felt like everything I thought and believed was pulled out from under me, and I was like oh, so that’s how it is! And now I can see that everything I’m thinking, feeling, and reacting to has to do with me and my background. It’s not objectively connected to what’s going on out there in the world. So, the part of DBT that worked the best for me was that […] it changed my way of thinking about my own contribution, and to take responsibility for my own contributions. What I do, think, and say and stop blaming others. Just not interpreting stuff into other people and situations.

Like Lisa, both Anna and Eva explained how DBT taught them to pause and try to figure out what was really happening in a situation and understand what other people were actually saying. This became a way for the participants to work against and overcome their tendency to misinterpret.

In addition to new perspectives on situations, the participants highlighted the value of the knowledge they gained about different emotions in DBT. In the interview Eva talked about the valuable experience of learning how one feeling can conceal another, such as anger concealing vulnerability. This enabled her to better understand and put into words how she felt. They spoke of an increased capacity to connect feelings to situations, which made their own feelings more understandable. Anna also emphasized the importance of knowing that feelings are not permanent. She said, “*Learning that it’s not dangerous even though it hurts, it will pass. It helped me a lot when I learned that [the feeling] will always pass. And I had not thought of that before, I was so preoccupied with how painful it was*.” Thus, explicit learning about feelings were central in the participants’ stories of change.

Furthermore, both Anna and Eva highlighted how information and knowledge about why they struggled made them more accepting toward themselves. Eva explained some important aspects of DBT, “*Understanding why I react as I do. Not feeling crazy, thinking there’s something wrong with you. And when there’s a reason and cause, you can do something about it. […] That made a huge difference. There’s hope not just hopelessness*.”

In this quote, Eva spoke of how knowledge about feelings and reactions helped her create a sense of meaning, and that this normalized her experiences. Furthermore, she demonstrated an ability to balance her focus between acceptance of herself and a hope connected to the possibility of change.

In combination with learning new information and knowledge, all four of the participants talked about the importance of the specific tools and skills they learned in DBT which enabled them to cope with emotions. This made them believe that it was possible to feel better. In the interview Sara said, “*It takes a while before you see, okay, I’m able to tolerate these feelings, and then you are maybe able to tolerate them for one, two, three minutes, and then you take a walk and you are suddenly gone for two hours*.” Similarly, Eva explained the tools she learned to use when she felt like harming herself, “*You postpone it a little. It’s like an impulse, the feeling is strongest in the beginning. After a while it’s easier to regulate when your head works a little. […] For me, counting something worked. It still does; it happens automatically*.” In this quote Eva explained how she could change the way she felt by using concrete techniques to regulate herself when emotions were strong. Some of the participants also highlighted the importance of practicing and repeating tools so that it was possible to use them automatically when needed.

Anna: I’ve practiced DBT every day since DBT. After a while it felt natural. You cannot just attend DBT skills training for a year and expect everything to be solved. Because the truth is, the difficulties you face when you have this diagnosis were probably created early in life, in most cases. They’re rooted deep inside you, so it takes a lot of hard work and repetition. And the more you practice the easier it gets.

Furthermore, the participants described how they developed a greater sense of agency. Lisa, Eva, and Sara all talked about how DBT taught them to believe that they could affect situations themselves. Lisa said, “*I experienced that more was up to me. Before, I felt like a victim of my circumstances. In DBT I learned that it was a consequence of how I see stuff, and react, it’s not necessarily related to what’s happening*.” In this quote Lisa explains how her experience of change in agency is closely linked to what she learned in DBT.

All the participants described how DBT was more helpful than previous treatments, and some also explained how it changed their lives. Eva and Anna talked about trying many different therapies and psychologists without much effect before DBT. In the interview Anna said, “*Before DBT everything was about therapy, hospitalizations, medication. Starting school, leaving, starting a job, quitting. […] I tried everything, and it wasn’t until DBT I noticed things got markedly better. I cannot say it enough, it has changed so much*.” In unison with Anna, Eva described how DBT helped her getting to know herself and how she acquired and learned to use concrete tools. She also spoke about how this format fitted her personality. One of Eva’s most important goals in treatment was to stop self-harming, and specific skills she learned in DBT helped her do this. This was one of the reasons why she felt like DBT worked. Lisa said she wished she had been referred to DBT earlier. Similarly, Sara talked about how DBT spared her of suffering: “*I do not know where I would have been without DBT. Still a lot of chaos*.” All four of the participants described how DBT seemed to represent an important turning point in their lives regarding their healing process.

As much as the participants emphasized the importance of learning tools, they differed in whether they found the specific ones helpful. Sara and Eva both described how they did not like mindfulness, because it only amplified the chaos of thoughts and feelings they had inside. In the interview Sara said, “*I think you should be more mindless […] it’s not that I need to be more present, sometimes you just need to shut everything out*.” In contrast to Eva and Sara, Anna described what mindfulness meant to her: “*Especially mindfulness has helped me a lot. […] Being able to take a pause, breathe for a second and try to see a situation for what it actually was.”* Anna further explained how she began to use mindfulness daily after DBT as a way of connecting to her inner states.

#### First meta-theme MBT: a long-lasting process of exploring to create procedural learning

The first meta-theme of the second topic for MBT seeks to understand the participants’ experiences of making sense of their world during treatment, and how this continued after treatment ended. One aspect that seemed important for all the participants was the integration of relational experiences, from past to present. By reaching an understanding of relational experiences and their own suffering, they also gained a sense of self-acceptance regarding themselves and their reactions. Amanda was able to understand that her experiences with domestic violence in her childhood and in her relationship gave her a certain perspective of the world. Similarly, Daniel developed a greater understanding of his childhood’s contribution to his functioning before MBT. Integration of past and present relational experiences thus seemed to have been a core focus during therapy and therefore became important aspects of the participants’ experience of change.

The participants also emphasized the importance of enough time for change to happen. Amanda talked about how it took several years for the changes to sit. Daniel pointed out how therapy is not a quick fix, but a way of unlearning your old habits and establishing new ones. Daniel said, “*If you walk the same route over and over, it’s easy. But if this route always leads you to a mountain wall, it’s not very productive. So, you have to make your own route. It’s really heavy, but when you walk that trail again and again, that one eventually also becomes easy*.” This quote demonstrates how hard and long-lasting a recovery process can be. However, it also says something about Daniel’s increased acceptance of his struggles, and his increased feeling of agency, because he realized he could do something about the status quo. He also said, *“It was not until the end I managed to reflect on how my anger affected others,”* which underlines the importance of time in the process leading up to such reflections. Even though Miriam received a shortened version of the MBT program, she also stressed the importance of time. This was particularly related to how establishment of trust and safety in the group can take time, and how recovery from BPD symptoms needs time.

Through the lengthy process described above, the participants spoke about how a greater understanding of their own feelings through an increased capability to mentalize were crucial elements in their healing. With increased mentalization came greater stability and understanding of self and others. Daniel talked about how therapy helped him deal with his pain, and how increased awareness of his struggles and feelings led to him gaining more stability in his daily life. He said, “*I connected with other feelings than anger, and to a certain degree I managed to be with the pain and my struggles. And I was assured that it was not dangerous to have such feelings*.” In the description of Daniel’s life before therapy, he talked about a life on a rollercoaster. About his life at present, he continued: “*I have my ups and downs, but my life is more stable, I’ve landed in myself. Most of the time I know my triggers, and my life is generally more stable.*”

Amanda talked about how a wider perspective of herself and others helped her recover. She emphasized how she slowly managed to widen her perspective in different situations. She said, “*Pausing between situations and the reaction. Thinking, what is happening, what am I feeling, what is she feeling, what did she mean. That process was non- existent before therapy. [My therapist and I] did not talk about it, but I believe that is the effect of mentalization*.” Amanda also described a troublesome relationship with one of the group therapists, however, she eventually realized that this person reminded her of someone else. Amanda said, “*It was helpful, understanding that my struggles with her had more to do with me than her. It was a good experience.”* This implied that Amanda gained a greater perspective of her own contributions to relationships with others, which she in turn ascribed to a greater ability to mentalize. In addition, the more she understood her own reactions and feelings, the more comprehensible others became.

Daniel also described how increased mentalization made him able to deal with situations that would have been hugely problematic before therapy. He said, “*Other people can have a really shitty day without saying anything. I understood how others’ negative or positive feelings did not necessarily have anything to do with what I had said or done.”* Miriam also gained a greater understanding of her attribution of feelings and thoughts to others, by looking at the way in which she interacted with others from a new perspective. She described this awareness of own patterns as an epiphany. During therapy she gained an understanding of how she was allowed to feel the things she felt, and that yelling at herself did not make the situation any better. Miriam’s increased tolerance and acceptance of own feelings also made her realize that she was a whole person who had needed the maladaptive coping mechanisms to deal with her illness.

Miriam: [Before therapy] I felt I had to give up my personality. But later [during therapy] I found out, the things I was doing were not my identity. I am still the same mum, the same wife. The way I am overthinking and ascribing feelings to others, it has nothing to do with my identity. It was just tools I needed in a period of my life to survive. It was an aha experience. I am not my struggles.

Daniel and Miriam, who both had ‘careers’ in psychiatry since childhood, described how MBT gave them something that previous therapies had not. They both emphasized how MBT enabled them to connect with their feelings. They both gained a sense of stability, that in turn enabled them to work through traumas and gave them hope for an actual recovery process from psychological struggles. Miriam also spoke about how MBT enabled her to begin a different treatment for her traumas. In addition to highlighting the positive sides of MBT, Daniel mentioned how he missed a focus on personal resources, and felt there was little room in the group therapy to talk about positive experiences. Amanda talked about how she missed a focus on her internal states, rather than just a focus on understanding others, and mentioned how she could have benefited from more explicit learning about emotions.

#### Second meta-theme DBT: a predictable program felt safe but less flexible

The second meta-theme of DBT explores the participants’ experiences with the structure of DBT as well as their experiences with their therapists and their groups. Both Eva and Sara talked about how the educational format of DBT was essential to their recovery process. Eva said, “*DBT gave me concrete things to go out and try*,” and Sara said, “*I do not think you can do it [recover] without the education, you need something systematic, you need to learn it on a child’s level*.” These quotes demonstrate to a certain extent how the participants valued the pedagogical format of DBT.

However, Eva, Sara, and Lisa explained that some aspects of the specific format of the DBT skills training were challenging. Eva described how she felt like the therapists in the group skills training could be inflexible about what worked for whom. Eva said, “*Not everything works for everyone. Like mindfulness, it did not work for me.*” Similarly, Sara experienced that the skills trainers occasionally presented advice as if behavioral change was easy. As a result, Sara felt that not all the advice from the group skills trainers were useful, when it was not adapted to the individuals in the group. Both Lisa and Sara explained how some of the other group members did not contribute to the group, either by not showing up or by being quiet. Lisa explained the consequence of participants not showing up: “*[…] I felt it ruined both the dynamic in the group and what I could get out of it when I had no one to work with. One time it was just me alone with two therapists, no one else showed up.*” Similarly, Sara described how the lack of participation from quiet group members affected her progress in the group because it made learning interpersonal skills difficult.

All the participants highlighted the importance of genuine therapists. Anna and Eva felt like their therapists cared for them. Anna spoke of her therapist’s importance for her recovery, “*Having a person who genuinely wishes the best for you, but at the same time sets boundaries and all that stuff… She means a lot to me*.” Lisa and Sara both highlighted the importance of how their therapists could share their reactions and emotions. About her individual therapist, Lisa said, “*I remember it was something I appreciated about her, when she said something, because I could see, wow the woman reacts, she has feelings!.*” Sara and Anna emphasized how their therapists felt predictable and safe. Anna described what she valued with her therapists: “*The fact that they were concrete, and that it was easy to understand what they meant made me trust them a lot. I knew they were there, and I knew they would tell me the truth.*”

#### Second meta-theme MBT: the therapist followed my lead, which made therapy relevant but challenging

The last meta-theme explores the participants’ experiences with the therapists and the group members. For Daniel and Miriam, a safe attachment and emotional bond to their therapists were valuable. Daniel emphasized how the therapists made use of his way of expressing himself, which in turn strengthened the emotional bond. He said, “*She gave me an image of my way of being near others. She said, “if you imagine a cactus in the desert, who wants to hug a cactus?,” and suddenly I realized how others perceived me*.” Daniel felt seen and understood by his therapist, who managed to tune into his use of language and ways of understanding the world. Miriam also talked about how the other group members became internal objects she could evoke to comfort her in times of pain, and it seemed like Daniel experienced an emotional correcting relationship in a therapeutic dyad that contained all parts of him.

Amanda, in contrast, often experienced the therapists as passive. For example, she experienced the group therapy as immensely intense and challenging, due to the group members’ combined emotional instability. She said, “*Everyone is unstable, it’s so volatile, because no one is stable, except the therapists, and you can feel the intensity and … it’s like matches always being in flames*.” For her, the therapists did not do enough to make the intensity bearable. Amanda also experienced that her individual therapist did not understand what she needed or took her concerns seriously enough. She perceived him as passive, and to a certain degree, dismissive, particularly regarding her experiences with domestic abuse.

## Discussion

### Different explanatory models for similar symptoms

The IPA indicated both similarities and differences in the groups of participants regarding how they understood themselves prior to treatment. One might argue that ways in which the participants described their lives before MBT and DBT could indicate how these stories became influenced retrospectively by the treatments they received. They all spoke about how life could be chaotic and overwhelming, which may be attributed to symptoms related to the diagnosis (41). However, differences could be seen in how they described their lack of coping mechanisms. The DBT participants spoke about not being able to change their mindset and how they felt. This may represent a way of thinking that is influenced by the cognitive behavioral tradition DBT springs out of, where working with changing thoughts is an important way of regulating emotions (85). In contrast, the MBT participants spoke about their lives before therapy from the perspective of the other, which may indicate a mentalizing stance (39, 86). There was also a striking contrast in the representation of self-harm in the participants’ stories. Most of the DBT participants provided detailed descriptions of self-harming experiences, whereas the MBT participants did not elaborate on the subject to such an extent. This may be a result of the focus on self-harm in DBT and MBT, respectively. The former uses skills training actively to replace self-harm as a coping mechanism (21), whereas the latter focuses less on such skills, and believes the frequency of self-harm will decrease with increased capacity to mentalize and reduced attachment avoidance (39).

Furthermore, participants in both groups highlighted a tendency to misinterpret others and sensitivity to rejection and judgment cues, however, this was described somewhat differently. The DBT participants were particularly concerned with not being able to perceive interactions with others and the world objectively. These descriptions may be influenced by DBT’s concept of “emotion mind,” where assumptions are made in an emotional state without checking the facts (85, 87, 88). The MBT participants rather emphasized not understanding their own and others’ inner states, in line with MBT’s focus on mentalization failures and psychic equivalence (22, 39). Some of the MBT participants also described how mentalization failure could lead to challenges with boundaries, inwards and outwards. MBT assumes that struggles with boundaries are linked to disturbance of mirroring in childhood (39). One could therefore argue that receiving MBT not only helped them form better relationships going forward, but also gave them a better understanding of why their past relationships had been so turbulent.

### Explicit and implicit ways of facilitating the change narrative

The participants’ stories of change as a result of treatment seemed to be told from a perspective situated in the therapeutic traditions they were socialized into. Elements from their stories represent both the theories of psychopathology and specific techniques used in the healing process. The DBT participants indicated explicit learning of a provided approach, and the MBT participants’ stories concerned a more implicit or procedural process of change.

The DBT participants spoke about learning to look at situations more objectively and understand their own contributions. DBT theory states that you can change an emotion if you change your interpretation of a situation to fit the facts (85). This enabled the DBT participants to understand how their interpretations were affected by emotions and their background. The DBT participants also spoke about the importance of the educational format, e.g., learning about emotions. This could be ascribed to the DBT skill of “observing, describing, and naming emotions,” and to a pedagogical approach to emotions in the individual therapy (21, 85). Furthermore, concrete tools to practice were highlighted as important for change. Thus, change in therapy might have been experienced through an explicit and educational learning process where new meanings were developed through acquiring the knowledge and tools offered by DBT.

The MBT participants, in contrast, emphasized the importance of having enough time for change. MBT proposes that being able to distinguish between fantasies and the real world takes time (39). Integration of past and present, and how early relational experiences may have affected them, were stated as important for change. This is aligned with MBT theory that an individual’s ability to interpret mental states and intersubjective transactions are affected by early attachment relations (39). Lastly, the MBT participants emphasized the importance of implicitly and procedurally developing and strengthening their ability to mentalize. One of the goals of MBT is to restore, maintain, and keep mentalization going when it would otherwise be lost (22). These differences converge with novel findings by Barnicot et al. (89) who studied patients’ experience of MBT and DBT for BPD. Among their findings it was proposed that learning emotion regulation skills and distress tolerance were uniquely characteristic of having received DBT while learning to mentalize in an interpersonal context was unique to experiences of MBT (89).

Both groups told a story of change highly influenced by the content of the treatments they received, but the process could be understood as different. One interpretation could be that the DBT participants acquired a map or learned an approach already made specifically for their problems, whereas the MBT participants created the map or explored new ways of moving forward. However, the differences between the two treatments are not clear cut. MBT is also a manualized treatment with concrete interventions (39, 90, 91), and automatization of skills in DBT (85) may create procedural learning. The difference might therefore be how DBT offers an explicit and declarative acquisition of knowledge and skills, and how MBT uses a more fluid and following exploration to create procedural learning in an implicit way (16, 21, 39, 85, 86).

The explicit and implicit procedures were also reflected in descriptions of how the therapeutic relationship could facilitate or challenge progress. This could reflect how the therapeutic relationship unfolds differently in different treatments (15, 16, 18, 92, 93). Research has indicated that an interaction between the specific and the common factors is needed for therapeutic change (17, 19). This has been elaborated by Ulvenes et al. (18) who proposed that the working alliance may operate differently in each therapeutic tradition. The significance of the working alliance might be common, but its interaction with a particular method may not be Ulvenes et al. (18). Research also suggests that treatments aimed at deeply rooted personality structures will often need a stronger emotional bond, compared to, for example, therapies targeting specific phobias through exposure (92, 93).

The DBT participants emphasized predictable and structured therapists and how this promoted a feeling of safety important to their healing process. However, some of the DBT participants experienced a lack of flexibility in the group. As the structure may be similar for most DBT groups, adapting tasks to specific participants may not be provided. This could be related to how some of the participants spoke about lack of attendance and participation from other group members, which they felt negatively affected the effect of the treatment. One interpretation could be that some participants experienced the topics as either unengaging or less relevant, hence participation might have suffered.

The MBT participants’ different experiences in the therapeutic relation might be related to the not-knowing position in MBT (94). One participant, Daniel, emphasized how the therapist followed his lead by speaking his language. Amanda, in contrast, perceived the therapists as passive and unauthoritative, contributing to a feeling of insecurity in treatment and intensity in the group. This could indicate a weaker working alliance. She still improved her ability to mentalize, which may imply BPD patients can experience change although the emotional corrective experience is not as powerful.

The findings from this study may indicate that in MBT-G, where group members may decide the agenda, the therapists can either seem too passive or like they follow the group members lead. The content of the group may either seem emotionally relevant or it can make the group feel unstable and uncontrolled. In contrast, the therapists in the DBT group may be perceived as both predictable, and more rigid and inflexible in the pedagogical format. Furthermore, the content of the group may either feel controlled and relevant, or irrelevant and unengaging. These results converge with Garred and Gough’s (16) findings indicating that a therapist’s project could be described along the dimensions from a *leading* (DBT) to a *following* (MBT) strategy, and from a *held* (DBT) to a more *fluid* (MBT) format. This could be understood considering the two therapeutic traditions that DBT and MBT springs out of, namely cognitive behavioral therapy and psychodynamic therapy (21, 51). In the cognitive behavioral tradition, it is more common for the therapist to explicitly educate the patient and rehearse new and concrete skills to handle or relieve the patient’s symptoms (95). In psychodynamic tradition the therapist, to a greater extent, seeks to follow the patient’s lead in developing a new understanding and gaining insights. In psychoanalysis, this is often referred to as evenly suspended attention or the analytic attitude (96, 97). Furthermore, Nilsson et al. (20) found that patients who had received cognitive therapy experienced their therapists as experts with concrete tools to teach in a structured and safe therapeutic setting, but that this setting also could be experienced as rigid. On the other hand, patients who had received psychodynamic treatment experienced their therapists as someone with whom they could explore past and present, and the therapists were experienced as more passive, which for some patients was a challenge (20).

The elements described above overlap with other qualitative studies investigating patients’ experiences of DBT or MBT (60, 61, 89, 98–104). These studies have accounted well for the various helpful elements in DBT and MBT, such as the quality of the working alliance and the usefulness of specific interventions. One may therefore argue that the separate elements demonstrating the effectiveness of these talking cures have been major suspects in the detective story about what makes psychotherapy work. However, the complete stories of change have to our knowledge been less investigated. These stories can shed light on how a cultural change narrative can develop as a result of psychological treatment and that such a narrative could be explicitly or implicitly transmitted from therapist to patient.

### Change through the internalization of a specific narrative

In mental health care a variety of psychotherapeutic methods are provided. In this study however, the participants were aware of the specific treatment in advance. One could therefore argue that the socialization into the treatment already began before the treatment started, and thereby the internalization of the cultural change narrative. This might have influenced the development of expectations (30). This is also evident in the interviews, where the participants consistently referred to treatment as either DBT or MBT, and previous treatment they received as just therapy. This might be due to the nature and focus of the project, but it could also reflect an unconscious affiliation or association with the method itself.

The development of expectations toward a treatment could also depend on the therapist’s ability to provide a believable rationale that is accepted by the patient. The rationale is one of the common factors highlighted by the CF approach (6, 29, 30). Several participants spoke about how DBT and MBT were more helpful than previous treatments. This may imply an acceptance of the rationale, and possibly that their expectations were met or exceeded. However, the cultural change narratives described indicated differences in the rationales provided by the treatments. In DBT one rationale could be the importance of addressing emotion dysregulation because it can lead to harmful behavior. Emotion regulation could be developed by practicing the ability to accept emotional states through mindfulness and distress tolerance (85). These skills were described by the DBT participants as important for recovery. In MBT, one assumes that the healing process relies on an increased capability to mentalize, and that increased ability to learn from interpersonal situations outside of treatment is facilitated through the therapeutic relationship (39). Increased capability to mentalize was even referred to as a superpower by one of the participants. These differences support the claim that the common factors, exemplified by the rationale, must be put into the context of the specific therapeutic techniques for them to be effective (105). Furthermore, it illuminates how two therapeutic methods with many similarities on the surface, could create differing descriptions of change on the experiential level of the individual patient receiving either MBT or DBT. These descriptions seem to convey the internalization of the specific culture that makes up DBT or MBT and thus the internalization of the cultural change narrative that each treatment offers.

The interaction between common and specific factors can also explain why different rationales for treating the same disorder could be equally effective. One explanation might be that the particular content is not of highest importance, but whether the rationale is believable, accepted and considered relevant to the patient. This could be seen in the light of epistemic trust, a concept embedded in the theoretical frameworks of MBT. The participants’ seemed to have considered the rationale’s information and knowledge about the world as relevant to them (106). One may therefore argue that epistemic trust could be important when a therapist conveys the rationale to a patient. To investigate this, one may look to Fonagy and Allison (107). They proposed several communication systems that contribute to developing epistemic trust, and possibly the ability to accept the treatment’s rationale.

Communication system 1 refers to the teaching and learning of content and system 2 refers to the re-emergence of robust mentalizing (107). According to this conceptualization, all treatments use communications system 1 to convey their rationale and for communicating that the therapist possesses knowledge and personal characteristics that could prove valuable to the patient (108). Based on our findings, it could be argued that DBT, to a greater extent than MBT, makes use of explicit teaching throughout the treatment as a way of fostering epistemic trust. This is because the rationale and its relevance for the patient, seems to have been communicated pedagogically. Although MBT also consists of psychoeducational groups, it could be argued that important elements of MBT are lacking in concrete and explicit operationalization (109). Explicit learning also seems less prominent in the MBT participants’ change narratives. However, recent studies of MBT have taken interest in developing an explicit pedagogical stance more clearly seen in DBT (38). Psychopedagogic interventions suited to MBT are suggested to be information about emotions, attachment, and social rules (38).

Communication system 2 concerns how therapists can aid the development of epistemic trust by exploring the content of the patient’s mind rather than offer new knowledge (107, 108). For example, if the therapist marks the patients experiences and emotional states, mentalizes the patient, and responds sensitively, “the patient takes a step back from epistemic isolation, and […] gradually begins to exercise his/her mentalizing skills” [(108), p. 9]. However, according to this conceptualization, “mentalizing is not its main goal, but the improved mentalizing that results from it enables the patient to start to approach and learn from their wider social context” [(108), p. 9]. Improved mentalizing, and thus an ability to learn from and consider the perspective of others, was emphasized in the MBT participants’ change narratives.

Despite the emphasis on mentalization in MBT, one could argue that some explicit techniques in DBT have overlapping qualities with communication system 2. For instance, the “wise mind” in DBT involves taking a pause and considering the possibility that other peoples’ intentions could be different from the patient’s own emotional mind (85, 88, 110). The DBT participants spoke about learning that their perception did not always coincide with the actual situation, suggesting that the DBT therapists explored and marked the patients’ emotions and experiences. This could have led to an understanding of how emotions affected their interpretations and reactions, which in DBT is referred to as “emotion mind” (85). One might therefore argue that “emotion mind” is similar to psychic equivalence in MBT (86, 88). Common features of DBT and MBT could thus be disguised by different language and treatment cultures but help patients in similar ways regardless. It also provides an example of how the common factors are put into context of the specific treatments. This overlap has led some authors to suggest incorporation of mentalization techniques in DBT to increase self-coherence, metacognition and attachment security (88). Even though epistemic trust is a concept primarily used in MBT, it could be useful when attempting to understand how patients accept a treatment’s rationale, and therefore be relevant to different schools of psychotherapy. Simultaneously, one could question the application of epistemic trust as a concept on the DBT participants’ experiences as it may compromise with established DBT theory.

The CF approach also stresses the importance of a culturally embedded explanation for the disorder that is being treated; an explanation in line with the dominant view of human experience in a given culture at a given time (6, 29, 30). An important part of the participants’ change narratives was being provided an explanation for why they struggled, facilitating normalization and acceptance. These different explanations were evident in the participants’ narratives of how they struggled prior to treatment. Although the explanations were different, both fit the dominant view of human existence in the present culture and time, and both led to acceptance and normalization.

The CF approach also highlights the importance of a therapeutic procedure or a healing ritual that promotes progressive behavior (6, 29, 30). The DBT participants easily expressed these procedures as it was communicated explicitly in therapy. It seemed harder for the MBT participants to formulate specifically what the treatment provided, but they pointed to how interactions with others changed as they increased their ability to take the other’s separate mind into account. This may reflect the development of a mentalizing stance.

The common features of psychotherapy, suggested by the CF approach (6, 29, 30), interacted with the treatment the participants received to create a cultural change narrative. These elements are all dependent on the therapist and the therapeutic relationship, which is regarded as particularly important in the treatment of BPD (70, 111). The working alliance was perceived differently both within and between the groups of participants. The variance ranged from the emotional bond being central for change to elements of the working alliance being strained. These results can be understood in light of Finsrud et al. (31) who propose that various measures of the working alliance related to change can be explained by two underlying factors, which in turn suggest two different pathways of change. The first pathway, *Confidence in the therapist*, suggests that patients do not differentiate between various therapist qualities, such as empathy and expertise. The second, *Confidence in the treatment*, is reflecting the patient’s experience of buying into the treatment. The latter could be related to the therapist’s ability to provide a believable rationale, which is accepted by the patient, and thereby create expectations and hope connected to the treatment ritual itself (6, 30).

Based on these findings one may argue that the participants who emphasized the emotional bond as important for change had confidence in the therapist. The participants who experienced the therapeutic relationship as difficult, but experienced change regardless, might have been on the second pathway, confidence in the treatment. This may imply that even though the bond was described as strained, the therapists seemed to convey a believable change narrative by tapping into the cultural healing myth of the treatments and linking this to expectancy and hope (6, 30).

To be able to convey confidence in the treatment, it seems central that the therapist believes the rationale they are providing. Studies on expectancy effects have suggested that treatment effects increase with the provider’s belief in the treatment (6, 33, 34, 38, 112, 113). This supports research suggesting that being anchored in a theoretical framework is associated with positive outcomes (114, 115). Considering the effects of placebo and expectancy one may argue that one central characteristic of expert therapists is that they believe in, and master, the treatment they provide. This is in line with our findings, where therapist characteristics became central aspects of the participants’ change narratives.

## Conclusion and limitations

This study suggests that DBT and MBT facilitate different cultural change narratives in their patients. The DBT participants spoke about the provision of an explicit approach, where concrete tools, techniques, and knowledge were offered, as well as a therapeutic relationship consisting of predictable and safe therapists. In contrast, the MBT participants spoke about a long-lasting process of procedural learning, focused on mentalizing abilities through exploration of past and present as well as describing a therapeutic relationship where the therapist followed the patient’s lead. Hence, the change narratives received by the two groups of participants seemed strongly influenced by the culture, myths, rationales, explanations, and procedures represented in the treatment they received. When considering the experienced impact of psychotherapy, the culturally embedded change narrative, and how it is created and conveyed, seems to strongly influence the impact of the received treatment. This is in line with the essential elements proposed by the CF approach. The specificity of the treatment the participants received provided a unique opportunity to research the joint effects of the methods provided and the therapist’s impact on the participants’ experiences of change. As this is one of the first studies that specifically target the cultural change narrative, further studies seem in strong need.

There are several limitations to this study that warrant further investigation. Firstly, IPA is interpretive in its nature and the findings of the present study will therefore both be a result of, and limited by, the authors’ pre-conceptions, values, and interests (82). With this in mind, multiple ways of analyzing the material are possible. There has been an attempt at transparency regarding the analytic path. The quotes will inevitably mirror a small sample of the whole material and other researchers might have been interested in different subjects. The limited number of participants, the unequal number of DBT and MBT participants, and the single male participant are other limitations. Lastly, we wish to address that several participants described experiences of severe stigmatization on account of their diagnosis in mental and somatic health care. We recognize this as an important issue to address, however, this was beyond the scope of the study. Future research on this topic is encouraged.

## Implications

The study has shed light on the significance of theories of change embedded in all psychotherapies and how this is conveyed and communicated. These findings seem to converge with the significance and interest therapists put into their clinical theories of change. This is because the cultural change narrative may become a core component of therapeutic change. Furthermore, it may suggest the strengths that lie in treatments and therapists that are able to offer a complete healing myth consisting of explanations, techniques and distinct ways to facilitate the therapeutic relationship. Therapists who accomplish this could benefit from placebo and expectancy effects. This may also relate to therapist affiliation, and allegiance effects, because if therapists believe in what they are offering, the healing myth may be more believable for the patient. Thus, it could be accepted and integrated into their stories of change. One could therefore argue that the most effective therapists are those who are allowed to provide the treatment they believe in. Furthermore, according to the Evidence-based practice in psychology (EBPP), patients have the right to receive treatment befitting for their characteristics, culture and preferences (116). This might support a pluralistic approach to the treatment of mental illnesses in public mental health care.

Additionally, a lack of pluralism could potentially limit the field of psychotherapy’s progress in research. This is because favoring one evidence-based treatment over another could hinder equal chances of funding (117). A pluralistic approach to the treatment of mental health also makes it easier for practitioners to learn from each other and from other schools of thought. Aspects of the present study suggest what MBT and DBT might learn from each other while highlighting the importance of the two treatments holding on to their respective healing myths. This could benefit the treatments and the therapists, but potentially also patients who will meet well-educated and well-informed professionals who have been exposed to a wide range of therapeutic methods, interventions, techniques and thinking (118, 119).

## Data availability statement

The original contributions presented in the study are included in the article/Supplementary material, further inquiries can be directed to the corresponding author.

## Ethics statement

The Norwegian Regional Committees for Medical and Health Research Ethics, National Center for Research Data and The Science Ombud at University of Oslo have approved the study. The participants received written and oral information about the project and its purposes. The participants signed a letter of consent before the interviews were conducted. They were free to end participation if desired. During the length of the project, careful consideration of the BPD patient group in general, and the participants in particular, has been important. Patients with BPD often experience stigma in psychiatric and somatic health care, and in society in general. Attending to the participants’ well-being as well as maintaining respect have been at the forefront of our consciousness at all times. The potential benefits of the study have been compared to potential costs. Measures have been taken to minimize risk throughout the whole project, but especially in the making of the interview guide and in carrying out the interviews.

## Author contributions

TS and AHT conducted the study and wrote the manuscript. ES and EF contributed with concept, design, conceptual issues, analysis, and edited the manuscript. All authors contributed to the article and approved the submitted version.
