# Supplementary material for: The cultural change narrative as a core component of therapeutic change
Source: Front Psychiatry. 2023 Oct 6;14:1149984. doi: 10.3389/fpsyt.2023.1149984 (PMC10587421; doi:10.3389/fpsyt.2023.1149984)
Supplement: Supplementary file 1 [file Table_1.docx]

**Appendix A: Tables**

**DBT**

| First topic: Before DBT | | |
| --- | --- | --- |
| **Meta-theme** | **Themes** | **Sub-themes** |
| I lacked an understanding of myself, and coped with my struggles destructively | I could not control my inner states | My feelings were overwhelmingly chaotic |
|  |  | No healthy tools to handle my feelings |
|  | Strategies to deal with overwhelming feelings | Self-harm to get rid of feelings |
|  |  | Self-harm to avoid being abandoned |
|  |  | Eating disorders to get rid of feelings |
|  |  | Withdrawal |
| First topic: Before DBT | | |
| **Meta-theme** | **Themes** | **Sub-themes** |
| My struggles with seeing the situation from an outside perspective | Sensitivity to others’ opinion of me | Fear of judgement |
|  |  | Shame related to other people’s perception of me |
|  | I misinterpreted other people | I was sensitive to rejection |
|  |  | I attributed meanings to others that they did not have |
| Second topic: During and after DBT | | |
| **Meta-theme** | **Themes** | **Sub-themes** |
| Explicit learning of a provided approach specific to my struggles | Learning new ways of understanding myself and others | Looking at the situation objectively |
|  |  | Learning about feelings |
|  |  | Working explicitly with self-acceptance and self-care |
|  | Learning new skills to handle my struggles | Learning new tools and repeat to automatize them |
|  |  | Agency |
|  |  | DBT more helpful than previous therapies |
|  |  | Differing experiences with mindfulness |
|  |  | Positive and negative sides of telephone coaching |
| Second topic: During and after DBT | | |
| **Meta-theme** | **Themes** | **Sub-themes** |
| A predictable program felt safe but less flexible | Lack of flexibility | The therapists could be more willing to adapt to the individual |
|  |  | Lack of participation from other group members |
|  | Importance of knowing where I had my therapist | Importance of having a plan for the therapeutic work |
|  |  | Importance of being genuine |
|  |  | The therapists could reveal something about themselves |
|  |  | Predictable and safe therapists |

**MBT**

| First topic: Before MBT | | |
| --- | --- | --- |
| **Meta-theme** | **Themes** | **Sub-themes** |
| My life lacked coherence | An incomprehensible self | Empty existence |
|  |  | A life in chaos |
|  |  | I concealed my pain |
|  | Harmful ways I dealt with my pain | Suicidal ideation/suicide attempts to handle feelings |
|  |  | Eating disorders as control over feelings |
|  |  | Vague descriptions of self-harm |
| First topic: Before MBT | | |
| **Meta-theme** | **Themes** | **Sub-themes** |
| How my problems with mentalization affected myself and others | Challenges with setting boundaries | Let others know |
|  |  | Disproportional aggression |
|  | Lack of mentalization | Misinterpretation of others |
|  |  | Lack of capacity for the other |
|  |  | The imagined other |
| Second topic: During and after MBT | | |
| **Meta-theme** | **Themes** | **Sub-themes** |
| A long-lasting process of exploring to create procedural learning | Integration of relational experiences | Self-acceptance through exploring how my past has shaped me |
|  |  | Changing how I perceive myself and the world through exploring takes time |
|  | I understand more of my feelings through an increased capability to mentalize | Feelings are more predictable and easier to handle |
|  |  | Increased ability to deal with my pain through a wider perspective of myself and others |
|  |  | MBT superior to other methods |
| Second topic: During and after MBT | | |
| **Meta-theme** | **Themes** | **Sub-themes** |
| The therapist followed my lead, which made therapy relevant but challenging | Positive outcomes related to the therapist’s not knowing stance | A safe attachment to the therapists |
|  |  | The therapists spoke my language |
|  | Challenges related to the therapist’s not knowing stance | The therapist was too passive and dismissive |
|  |  | Emotional intensity made the group therapy exhausting |
